# MLR Data-Driven for the Prediction of Infinite Dilution Activity Coefficient of Water in Ionic Liquids (ILs) Using QSPR-Based COSMO Descriptors

**DOI:** 10.1021/acs.jcim.4c02095

**Published:** 2025-02-21

**Authors:** Ali Ebrahimpoor Gorji, Juho-Pekka Laakso, Ville Alopaeus, Petri Uusi-Kyyny

**Affiliations:** School of Chemical Technology, Department of Chemical and Metallurgical Engineering, Research Group of Chemical Engineering, Aalto University, P.O. Box 16100, FI- 00076 Aalto, Finland

## Abstract

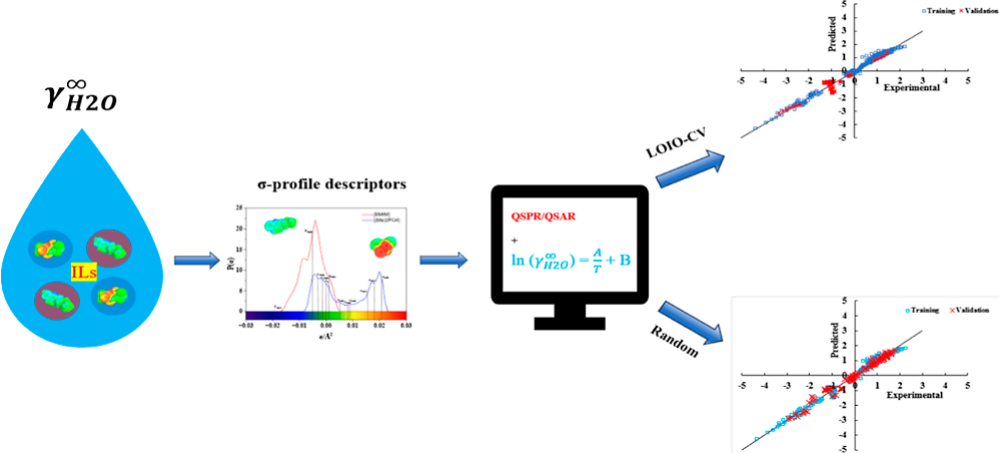

To predict the partial molar excess enthalpy, entropy at infinite dilution, and phase equilibria, the availability of an infinite dilution activity coefficient is vital. The “quantitative structure-activity/property relationship” (QSAR/QSPR) approach has been used for the prediction of infinite dilution activity coefficient of water in ionic liquids using an extensive data set. The data set comprised 380 data points including 68 unique ILs at a wide range of temperatures, which is more extensive than previously published data sets. Moreover, new predictive QSAR/QSPR models including novel molecular descriptors, called “COSMO-RS descriptors”, have been developed. Using two different techniques of external validation, the data set was divided to the training set for the development of models and to the validation set for external validation. Unlike former available models, internal validation using leave one/multi out-cross validations (LOO–CV/LMO–CV) and Y-scrambling methods were performed on the models using statistical parameters for further assessment. According to the obtained results of statistical parameters (*R*^2^ = 0.99 and *Q*^2^_LOO–CV_ = 0.99), the predictive capability of the developed QSPR model was excellent for training set. Regarding the external validation, other statistical parameters such as AAD = 0.283 and AARD % = 30 were also satisfactory for the validation set. While the values of γ_H_2___O_^∞^ increase or decrease with increasing temperature, the QSAR/QSPR models based on the van’t Hoff equation takes into account the negative and positive effects of temperature on the γ_H_2___O_^∞^ in ILs well, depending on the nature of ILs. It was also shown that γ_H_2___O_^∞^ in some new ILs which had not been experimentally studied before can be predicted using the QSPR model.

## 1 Introduction

Simultaneous presence of water (H_2_O) as one of the unavoidable associated compounds alongside natural gas streams may cause some serious issues such as freezing and hydrate formation.^1,2^ Hence, many researchers have been looking for finding a proper solvent to remove the water in the dehydration processes.^1,2^ Many conventional solvents such as ethylene glycol, diethylene glycol, and new solvents like ionic liquids (ILs) have been screened for the removal of water from natural gas streams.^1^

Among the solvents screened, ILs have recently received significant attention. ILs consist of two separate parts called “organic or inorganic anion” and “organic cation”. Some intrinsic and outstanding features of ILs, such as minimal volatility, high degradation temperature, high selectivity, and low flammability, caused the ILs have gained the growing interest for use in different chemical processes.^1^ When it comes to design and selection of appropriate ILs, the extensive combination of anion and cation imposes time-consuming and cost problems and restrictions for the engineers. Moreover, a molecular insight of their phase behavior and their interactions with other components like H_2_O is still required.

Infinite dilution activity coefficient (γ^∞^) is a key property for the prediction of phase equilibrium. γ^∞^ is one of the significant thermodynamic properties which has been frequently studied in the binary mixture of IL and H_2_O.^1,2^ Since γ^∞^ can provide helpful information on enthalpy and entropy changes and chemical potential, it can be considered as a good criteria and measure for finding the most suitable ILs with highest and lowest affinities into the H_2_O.^3^ The ln (γ^∞^) can be split into its respective entropic and enthalpic components.^3^ γ^∞^ values also show the interaction between solvent and solute.^4^ In other words, the higher the solute–solvent interaction, the lower the activity coefficient and vice versa.^4^ There are many commonly used methods for determining infinite dilution activity coefficent of compounds, such as gas–liquid chromatography (GLC) and vapor–liquid equilibria (VLE) methods.^5^ Computational^1,2^ and experimental [4,6–61] point of views, the influence of structural variations of ILs (anions and cations) on the infinite dilution activity coefficent of H_2_O (γ_H_2___O_^∞^), has been frequently studied by researchers.

Domanska and Laskowska.^6^ as one of most active research groups in this field measured the values of γ_H_2___O_^∞^ in many ILs. There are many experimental studies [6–61] including a large number of cation and anion structural variations. However, with an estimate of 10^18^ potential ILs,^3^ determining γ_H_2___O_^∞^ values for all systems is not practically possible. Hence, reliable white-box and predictive methods are of utmost importance. In the earlier computational studies, Gonfa et al.^2^ figured out that the COSMO-RS model as a direct predictive tool overestimates the γ_H_2___O_^∞^ in ILs. For this reason, they^1^ tried to apply and combine two computational methods which called “quantitative structure–property (or activity) relationship (QSPR/QSAR)” and “COSMO-RS”. Gonfa et al.^1^ suggested the following linear regression technique for the prediction of γ_H_2___O_^∞^ in ILs using new molecular-inputs (descriptors) and temperature (eq 1)
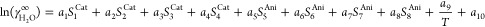
1where *a*_1_ to *a*_8_ are the adjustable parameters of the effect of structural variations (i.e., cation and anion) and *a*_9_ is the adjustable parameter of the effect of temperature (*T*) on the γ_H_2___O_^∞^ in the ILs. Equation 1 represents the relationship between the γ_H_2___O_^∞^ and independent inputs, which are the numerical values of the σ-profile descriptor and the temperature. They^1^ divided the sigma profile of each cation and anion into four regions. In other words, the occupied areas of each region are considered as COSMO-RS numerical molecular descriptors, called “*S*_1_^Cat^, *S*_2_^Cat^, *S*_3_^Cat^, *S*_4_^Cat^” and “*S*_5_^Ani^, *S*_6_^Ani^, *S*_7_^Ani^, *S*_8_^Ani^”. They have taken into account the temperature effect on the γ_H_2___O_^∞^ in the ILs, according to the van’t Hoff equation. The proposed model by Gonfa et al.^1^ never can consider the dependency of the temperature for all kinds of ILs. Because such model always has a constant adjustable parameter for “1/*T*” variable (i.e., *a*_9_). Although the γ_H_2___O_^∞^ in majority of ILs decreases with an increase in temperature,^62^ there are also ILs where γ_H_2___O_^∞^ increases or stays almost constant. For this reason and to avoid the deficiency of the model, Gonfa et al.^1^ presented separate linear QSAR models for each constant temperature. In the continuation of above study, Benimam et al.^63^ attempted to enhance the prediction capability using the nonlinear method called “Dragonfly-Support Vector Machine”. In their studies, they used the same kind of molecular inputs and temperature to model the γ_H_2___O_^∞^ in imidazolium-based ILs. To the best of our knowledge, there are only three research studies^1,2,63^ for the prediction of γ_H_2___O_^∞^ in ILs. However, there were some missing points in their studies which should be studied more. One same missing point in these studies was that all used limited amount of data points and limited variations of cation and anion. Another missing point which was quite obvious in a study by Gonfa et al.^1^ is the misunderstanding of 1/*T* term in van’t Hoff equation. To avoid of such misunderstanding, Thangarajoo et al.^3^ improved the use of the van’t Hoff equation by the varied coefficient of “1/*T*” term which was rationally depended on the molecular inputs for the first time. According to the proposed model by Thangarajoo et al.^3^ which was developed for methanol and IL mixtures (not γ_H_2___O_^∞^), the values of molecular descriptors of “1/*T*” vary with respect to the nature of ILs. Nevertheless, the molecular variables selection had been ignored in all three discussed studies.^1,3,63^ Therefore, applying artificial intelligence (AI) methodology as a predictive tool is still required to not only precisely adjust the varied parameters of the “1/*T*” term in the van’t Hoff equation, with respect to the structure and nature of ILs, but also select the proper molecular variables, simultaneously.

The (QSPR/QSAR) approach as one of the robust methods is commonly used to make qualitative and quantitative correlation between ILs and their specific properties.^64−67^ Therefore, it is possible to design and/or develop new ILs using the QSPR method for different applications. Up to now, researchers have not applied the QSPR/QSAR approach, including variable selection for the prediction of γ_H_2___O_^∞^ in ILs, with a large space of cation and anion structural variations at a wide range of temperatures. This approach has been applied in the well-known software called “QSARINS”^68−70^ as the first time which features several methods such as molecular variables selection, internal, and external validations in its environment. However, Gonfa et al.^1^ and Benimam et al.^63^ used a somewhat similar approach with this work but with a major difference with the lack of molecular variables selection. In this work, molecular variable selection has been carried out from a descriptors pool. A comparison with the proposed models by Gonfa et al.^1^ and Benimam et al.^63^ was carried out to highlight the improvements and novelty made in this study. This work has the novel feature of examining the advantages of using the QSPR/QSAR approach-based new COSMO-RS descriptors for the prediction of γ_H_2___O_^∞^ in ILs. Gathering and using a comprehensive data set [a much larger data set (i.e., 380 total data points)] including additional structural variations of cation and anion is one of the biggest differences in comparison with former data sets. The use of this new data set enables the development of the predictive models for a wide range of ILs. The developed predictive and reliable QSPR/QSAR model aids the chemical engineering community to select the proper ILs for the specific applications. In this work, we report in addition to γ_H_2___O_^∞^ the two most important thermodynamic properties [i.e., the partial molar excess enthalpy (i.e., ΔH_IL_^E,∞^) and entropy (i.e., ΔS_IL_^E,∞^) at infinite dilution] for numerous systems including IL and H_2_O to screen the ILs.

## 2 Methods

### 2.1 Basic Theory

In this study, the influence of temperature on the γ_H_2___O_^∞^ in ILs was included as an independent variable based on the van’t Hoff equation. Van’t Hoff equation expresses the relationship between γ_H_2___O_^∞^ in ILs and temperature as below (eq 2)
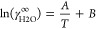
2where “*A*” and “*B*” are adjustable parameters which depend on the molecular descriptors and their weights determined by the QSPR method.

**Figure 1:**
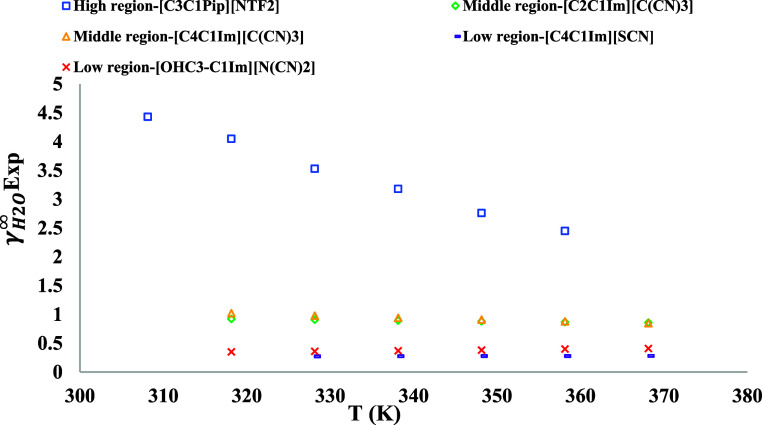
Examples of ILs from each region with different dependencies to temperature.^6,9,44,47,61^

The partial molar excess enthalpy (i.e., ΔH_IL_^E,∞^) and entropy (i.e., ΔS_IL_^E,∞^) at infinite dilution, which are shown in eq 3, can be calculated using “*A*” and “*B*” (see eq 2), respectively.
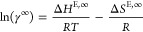
3In this study, the most significant COSMO-RS descriptors (points of specific charge densities) were applied to distinguish the effect of cation and anion structural variations. In fact, these prominent descriptors are rationally and strongly related to the two most important thermodynamic properties (i.e., Δ*H*^E,∞^ and Δ*S*^E,∞^). As mentioned earlier, the influence of the temperature on γ_H_2___O_^∞^ in the ILs varies with the nature of the ILs. For a majority of ILs, the γ_H_2___O_^∞^ value decreases as the temperature increases, but for a limited amount of ILs, the opposite behavior has been observed. Therefore, the predictive QSPR/QSAR based-COSMO-RS model should be able to consider this complexity. Hence, the model should be able to change sign of “*A*” parameter according to the behavior of the solute in the ILs. It means that the sign of “*A*” parameter is often “+”, stating the values of γ_H_2___O_^∞^ in the majority of ILs decrease. Otherwise (contrarily), the sign of “*A*” parameter is rarely “–”, stating the values of γ_H_2___O_^∞^ in other ILs increase. In brief, the included ILs in this study belong to three different regions with increasing temperature: (1) the values of γ_H_2___O_^∞^ decrease, (2) the values of γ_H_2___O_^∞^ increase, and (3) the values of γ_H_2___O_^∞^ are almost constant (see Figure 1).

As can be seen in Figure 1, three distinct regions can be categorized for the values of γ_H_2___O_^∞^ in ILs, called “high”, “middle”, and “low” regions. In other words, the values of γ_H_2___O_^∞^ in the high region decrease significantly with increasing temperature, and the values are relatively high or much higher than 1. While the values of γ_H_2___O_^∞^ in middle and low regions are almost constant (with very slight reduction) and increase with increasing temperature, respectively. The values of γ_H_2___O_^∞^ in the middle region are scattered around 1, and the values of γ_H_2___O_^∞^ in the low region are relatively low or much lower than 1. Some representatives of ILs for each region can be found in the Supporting Information Excel file (see Sheet S1). As briefly shown in Figure 1, H_2_O in [C3C1Pip][NTF2] belongs to the first region, where γ_H_2___O_^∞^ decreases remarkably with increasing temperature. H_2_O in [C2C1Im][C(CN)3] and [C4C1Im][C(CN)3] belongs to the middle region, where γ_H_2___O_^∞^ is nearly temperature invariant with increasing temperature. H_2_O in [C4C1Im][SCN] and [OHC3–C1Im][N(CN)2] is example of the third region, where γ_H_2___O_^∞^ increases with increasing temperature. Regarding the third region, it should be mentioned that the changes of values of γ_H_2___O_^∞^ in some ILs such as [C4C1Im][SCN] and [OHC3–C1Im][N(CN)2] are slight, while in other ILs like [C4C1Im][Br], changes can be tangible. These observed complexities of different behaviors of ILs make the modeling of such systems challenging, and novel sophisticated models are needed. Therefore, there is still a need to applying new methodologies on eq 2 (i.e., van’t Hoff equation), for further/future investigations.

### 2.2 Data Set

According to the collected experimental data [6–61], an extensive data set with very high structural variations of cations and anions has been created for QSPR/QSAR studies. The details of this data set can be found in Table 1 and Supporting Information Excel file (see Sheets S2 and S3). All in all, 380 data points including 68 unique ILs (34 cations and 15 anions) at a wide range of temperatures with the above-described features are listed in Table 1.

**Table 1:** Studied ILs in Different Binary Systems (i.e., IL + H_2_O) with Their Ranges of Temperature and Experimental Values as Well as Statistical Parameters

mixture IL/H_2_O	temperature range (K)	range of ln (γ_H_2___O_^∞^_Exp_)	AARD %^b^	refs
**[C4C1Im][SCN]**^a^	328–368	(−1.298) – (−1.269)	32.9	(6)
[C4–3-C1Py][CF3SO3]	318–358	(−0.116) – (−0.267)	73.4	(7)
[C4C1Pyrro][FAP]	318–368	(2.236) – (1.350)	8.4	(8)
**[C3C1Pip][NTF2]**	308–358	(1.488) – (0.896)	12.2	(9)
[HOC3Py][FAP]	308–358	(0.974) – (0.548)	26.3	(10)
[C4C1Im][TFA]	333–393	(−1.851) – (−1.639)	5.3	(11)
**[C2C1Im][SCN]**	328–368	(−1.298) – (−1.269)	30.5	(12)
**[C4C1Pip][SCN]**	328–368	(−1.105) – (−1.064)	25.6	(13)
**[C4–4-C1Py][SCN]**	328–368	(−1.187) – (−1.155)	24.7	(14)
**[C4C1Pyrro][SCN]**	328–368	(−1.335) – (−1.269)	37.1	(14)
**[C6C1Im][SCN]**	328–368	(−0.572) – (−0.623)	33.1	(15)
**[C6-iqui][SCN]**	328–368	(−0.913) – (−0.872)	17.1	(16)
[C4C1Mor][C(CN)3]	318–368	(0.029) – (−0.248)	315.9	(17)
[C4C1Pyrro][C(CN)3]	318–368	(−0.027) – (−0.261)	132	(18)
[C4C1Pyrro][B(CN)4]	318–368	(0.765) – (0.246)	6.9	(19)
[C10C1Im][B(CN)4]	328–378	(0.751) – (0.300)	4.6	(20)
[C2C1Im][B(CN)4]	298–358	(0.806) – (0.254)	1.6	(21)
[C6C1Im][B(CN)4]	318–368	(0.717) – (0.285)	3.4	(22)
[C4C1Pyrro][CF3SO3]	318–368	(−0.145) – (−0.339)	19	(23)
[C4C1Im][CF3SO3]	328–368	(−0.212) – (−0.362)	13.1	(24)
[C2C1Im][TFA]	348–368	(−1.944) – (−1.838)	10.0	(25)
[C6OC1C1Im][NTF2]	298–358	(1.474) – (0.712)	2.4	(26)
[(C6OCH2)2Im][NTF2]	298–368	(1.488) – (0.593)	6.7	(26)
[C4–4-C1Py][NTF2]	298–368	(1.581) – (0.717)	10.1	(4)
[N1112OH][NTF2]	318–368	(0.270) – (−0.069)	355.6	(27)
[C8iquin][NTF2]	328–368	(1.398) – (0.838)	5.7	(28)
[C4C1Pip][NTF2]	308–358	(1.566) – (0.879)	10.7	(29)
[S222][NTF2]	308–368	(1.363) – (0.631)	4.2	(30)
[C1C1Im][NTF2]	303–333	(1.050) – (0.698)	27.6	(31)
[C2C1Im][NTF2]	293–323	(1.302) – (0.932)	16.7	(31)
[C2Py][NTF2]	303–323	(1.022) – (0.693)	26.9	(32)
[HOC3Py][NTF2]	318–378	(0.425) – (0.029)	101.7	(33)
[COC2C1Pip][NTF2]	318–368	(1.208) – (0.625)	1.8	(34)
[COC2C1Mor][NTF2]	318–368	(0.912) – (0.343)	14.6	(35)
[COC2C1Pyrro][NTF2]	318–368	(1.147) – (0.615)	1.4	(36)
[C5C1Pip][NTF2]	308–358	(1.619) – (0.892)	10.3	(37)
[C6C1Pip][NTF2]	308–358	(1.669) – (0.896)	9.1	(37)
[C4–4-C1Py][N(CN)2]	338–368	(−1.224) – (−1.158)	33.2	(38)
[COC2C1Mor][FAP]	318–368	(1.547) – (1.101)	4.4	(39)
[COC2C1Pip][FAP]	318–368	(1.954) – (1.308)	4.4	(40)
[COC2C1Pyrro][FAP]	318–368	(2.086) – (1.371)	7.5	(41)
[HOC2C1Im][FAP]	318–368	(0.797) – (0.570)	16.5	(42)
[C1C1Im][DMP]	363–383	(−2.960) – (−2.856)	1.5	(32)
[C4C1Im][Br]	333–393	(−2.631) – (−2.017)	4.9	(11)
[C2C1Im][MeSO3]	318–358	(−2.645) – (−2.430)	6.7	(43)
[C2C1Im][C(CN)3]	318–368	(−0.080) – (−0.153)	41.7	(44)
[C2C1Im][FAP]	318–368	(1.906) – (1.425)	4.9	(45)
**[HOC2C1Im][N(CN)2]**	328–358	(−0.946) – (−0.991)	66.1	(46)
[C4C1Im][Ac]	298–393	(−4.342) – (−3.194)	1.2	(11)
[C4C1Im][C(CN)3]	318–368	(0.019) – (−0.164)	23.5	(47)
**[C4C1Im][Cl]**	333–428	(−3.324) – (−2.364)	3.6	(11,48)
**[C4C1Im][N(CN)2]**	318–368	(−1.171) – (−0.978)	25.2	(49)
[C4C1Im][NTF2]	293–363	(1.373) – (0.688)	6.7	(31,50)
[C4C1Im][DMP]	333–428	(−3.381) – (−2.292)	4.1	(11,48)
[C4C1Im][MeSO3]	333–428	(−2.120) – (−1.431)	8.2	(11,48)
[C6C1Im][CF3SO3]	303–333	(0.270) – (−0.446)	200.8	(51)
[B Cya Py][NTF2]	308–368	(1.196) – (0.693)	6.9	(52)
[C12C1Im][NTF2]	318–368	(1.501) – (1.022)	5.2	(53)
[C4C1Py][C(CN)3]	318–368	(−0.029) – (−0.228)	42.8	(47)
[4OHC3 4-C1Mor][NTF2]	318–368	(0.357) – (0.095)	48.1	(54)
[C8Quin][NTF2]	313–353	(1.071) – (0.350)	92.9	(55)
[C6Quin][NTF2]	313–353	(1.446) – (1.098)	6.9	(55)
[P4442][DEP]	328–368	(−2.292) – (−2.154)	9.1	(56)
[C8C1Im][NTF2]	303–333	(1.358) – (0.947)	13.3	(57)
[C2C1Im][C8SO4]	333–358	(−1.832) – (−1.897)	5.5	(58)
**[C2C1Mor][N(CN)2]**	318–368	(−1.127) – (−0.941)	0.95	(59)
[HOC3Py][N(CN)2]	328–358	(−0.918) – (−0.839)	74.6	(60)
**[OHC3–C1Im][N(CN)2]**	318–368	(−1.049) – (−0.891)	33.4	(61)

a Bold means validation set.

b Using eq 16 [see Supporting Information Excel file (see Sheet S4)].

### 2.3 Former Available Models

There were multilinear-regression (MLR) and nonlinear models for the prediction of γ_H_2___O_^∞^ in the ILs which were proposed by Gonfa et al.^1^ and Benimam et al.,^63^ respectively. The quantitative and qualitative descriptors were sigma profile segments of cations and anions. Although they used the small data sets with low variations of cations and anions for training and validation models, the results of this study will be compared with their results. They evaluated the prediction capability of their predictive models using coefficient of determination (i.e., *R*^2^) and root-mean-square error (i.e., RMSE %) statistical parameters. For this reason, *R*^2^ and RMSE % alongside other relevant statistical parameters will be reported in this study, for further comparisons.

### 2.4 QSPR Method

#### 2.4.1 Calculation of COSMO-Based Molecular Descriptors

Molecular descriptors are from σ-profile which is calculated via COSMO-RS theory.^71−74^ Sigma profiles are taken from database (COSMObase 2023) which is provided in COSMOtherm 2023^75^ software, and the chosen sigma profiles were calculated by using TZVP-basis set. Descriptor values are taken from the lowest energy configuration of cation and anion molecules.

**Figure 2:**
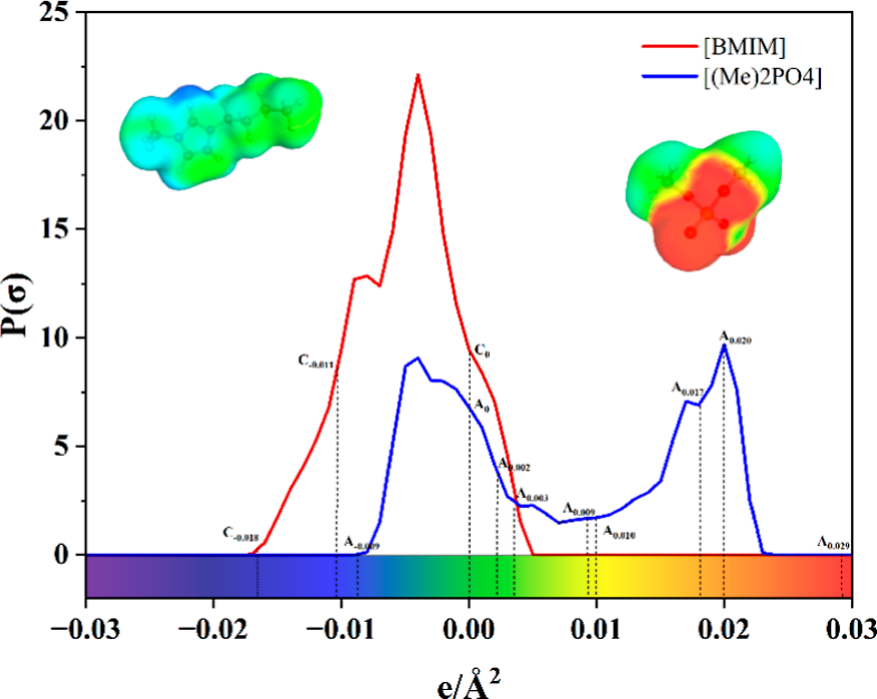
Demonstration of some cationic and anionic descriptors [i.e., values of probability (amount) at some specific charge densities (SCD)] for [C4C1im] cation (or [BMIM]) and [DMP] anion (or [(Me)2PO4)].

In a nutshell, sigma profile is 2D representation from 3D surface polarities of molecules, as shown in Figure 2, the *x*-axis tells you the strength of surface charge density (SCD) and the *y*-axis tells you the probability (amount) of finding this SCD. Sigma profile is calculated with 0.001 interval, and typically it varies from −0.03 to 0.03 e/Å^2^. An example of sigma profile descriptors for cations and anions is shown in Figure 2.

#### 2.4.2 Model Development

As can be seen in eq 2, ln (γ_H_2___O_^∞^) is a function of cation and anion descriptors along with temperature. Since both cation and anion structures were changing in data set, “A” and “B” including cation and anion descriptors must distinguish the effect of cation and anion structures on ln (γ_H_2___O_^∞^). For model construction, the suitable descriptors must be selected [i.e., the missing effort in the former studies^1,63^ (i.e., variables selection)] from 61 sigma profile descriptors. There are well-known methods of variables selection such as genetic algorithm (GA) method,^76^ artificial neural network (ANN),^77^ and replacement method (RM).^78^ In this study, GA was used to build MLR QSPR model-based COSMO descriptors. The details of the GA-MLR algorithm can be found elsewhere.^79,80^ QSARINS software was applied to develop the GA-MLR models.

#### 2.4.3 Statistical Parameters

The goodness-of-fit of the QSPR model should be carefully checked using the standard statistical parameters, including coefficient of determination (*R*^2^), leave-one-out cross-validated coefficient of determination (*Q*^2^_LOO_-_CV_), adjustable coefficient of determination (*R*^2^_Adj_), average absolute relative deviation (%AARD), average absolute deviations (AAD), Fisher function (F), root-mean-square error (RMSE), standard residual (*S*), and maximum (or critical) leverage (h*). More detailed information regarding the statistical parameters used in this study can be found in Table 2 (eqs 4–12).

**Table 2:** Applied Statistical Parameters in This Study^a^

introduced parameters	introduced parameters equations
coefficient of determination	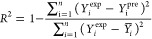 4
adjustable coefficient of determination	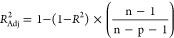 5
leave-one-out cross-validated coefficient of determination	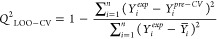 6
Fisher function	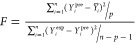 7
standard residual	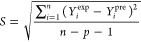 8
root-mean-square error (RMSE)	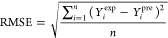 9
average absolute deviation	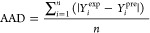 10
average absolute relative deviation %	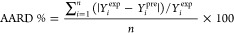 11
maximum leverage	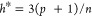 12

a *Y*_*i*_^exp^, *Y*_*i*_^pre^, , n, and p demonstrate experimental values (ln-Based), predicted values (ln-Based), average experimental values (ln-Based), the number of the experimental data set, and the number of employed descriptors, respectively.

Applicability domain (AD) analysis as a vital concept of the QSPR approach should be considered. It allows:^81^ (1) the uncertainty in prediction and (2) the extent of extrapolation of QSPR models.^82,83^ In order to predict γ_H_2___O_^∞^ in new ILs, it is essential that new ILs lie within the same AD space. In other words, it means that new ILs are physicochemically, biologically, or structurally similar to molecules used for model development (i.e., training set). The more space of AD, the more reliable predictions of the new ILs. To carry out the external validation using validation set, it is essential to ensure that the validations set of molecules is inside of QSPR model’s AD.^84^

The space of AD can be specified using two main parameters: (1) the leverage values (*h*_*i*_) and (2) the standardized residual (SDR) and. SDR was defined as eq 13
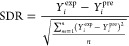
13where *h*_*i*_ represents a measure of a molecule’s distance from the center of the training set. It is needed to determine whether new ILs are within the applicability of the domain of the developed QSPR model or not. The parameter can be calculated with eq 14.
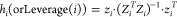
14where *z*_*i*_, *Z* are the descriptor row vector of point *i* and *a n* × *p* matrix of descriptors for compounds derived from the training set, respectively. AD of developed QSPR models can be obtained in QSARINS software for each model, and maximum leverage (i.e., h*) can be calculated using eq 12.

#### 2.4.4 Internal and External Validations

After building of the QSAR/QSPR model, it is essential to conduct internal and external validations on the training (approximately 75% of main data set) and validation (approximately 25% of main data set) sets, respectively. Regarding the external validation, the prediction capability of developed QSAR/QSPR models was evaluated using two validation sets which are created in two different methods:^85^ 1: leave random data points out-cross validation and 2: leave one ion out-cross validation (i.e., LOIO–CV). In the random method, some data points (not necessarily all) of ILs at some temperature may be set into the validation set, randomly. While, in the ion method, all data points of the specific cation and anion must be set into the validation set, completely. In the LOIO–CV method, neither the cation nor the anion of some ILs of the validation set can reappear in the training set. The LOIO–CV method enhances the stability of developed QSAR/QSPR models when predicting the properties of ILs with novel cations and anions, which is crucial for the data-driven design of new ILs.^85^

Regarding the internal validation, Y-Scrambling, leave multiout-cross-validation (LMO-CV), and leave one-out-cross-validation (LOO-CV) methods should be conducted on the developed QSAR/QSPR model. In fact, these methods were performed on the training set (not the validation set).

## 3 Results and Discussion

### 3.1 Developed QSAR/QSPR Models

Before the development of the QSAR/QSPR models, some ILs were set intentionally aside in the validation set (see Table 1, bold ILs). The LOIO-CV method guarantees the presence of ILs with new structures for both cations and anions. In another words, all present data points of those ILs including anions ([SCN], [N(CN)2]), and [Cl] or cations ([C4–3-C1Py], [C3C1Pip], [C6-iqui], [C2C1Mor], and [OHC3–C1Im]) in the data set were deliberately set aside into validation set. Among specified ILs as the validation set, the involved structures for both cation and anion in [C6-iqui][SCN], [C2C1Mor][N(CN)2], and [OHC3–C1Im][N(CN)2] were absent in the training set. The details of the validation set for the LOIO–CV method can be found in Supporting Information Excel file (see Sheet S5). The main aim of this categorization is to investigate the QSAR/QSPR’s prediction capability for new structures of cation and anion.

After dividing an extensive data set into training and validation sets for performing internal and external validations, the QSAR/QSPR model eq 16 which was developed using training set with above features is indicated in Table 3. Moreover, another QSAR/QSPR model eq 17 using another training set, which was created using leave random data points out-cross method, was built for further investigations and comparisons. The details of the validation set for the random method can be found in Supporting Information Excel file (see Sheets S6 and S7).

**Table 3:** Developed QSAR/QSPR Models Using Two Different Methods of External Validations (i.e., LOIO–CV and Random)

external validations	number of data points in training set	models^c^
LOIO–CV	297^a^	ln (γ_H2O_^∞^) = 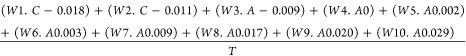 16 + (*W*11.C0)+(*W*12.A0.010)+(*W*13.A0.017)+(*W*14)
random	285^b^	ln (γ_H2O_^∞^) = 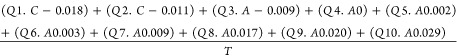 17 + (*Q*11.C0)+(*Q*12.A0.010)+(*Q*13.A0.017)+(*Q*14)

a 83 data points were set aside into validation set.

b 95 data points were set aside into validation set.

c Models’ parameters: *W*1 = −240.0077, *W*2 = −11.3664, *W*3 = −28.5232, *W*4 = −28.9690, *W*5 = 51.8990, *W*6 = −26.6256, *W*7 = 82.5691, *W*8 = −70.7223, *W*9 = −68.4551, *W*10 = −1816.7257, *W*11 = 0.0102, *W*12 = −0.1697, *W*13 = 0.1319, *W*14 = −0.4159 and *Q*1 = −166.4001, *Q*2 = −10.9408, *Q*3 = −42.8764, *Q*4 = −23.3850, *Q*5 = 53.5780, *Q*6 = −28.7002, *Q*7 = 88.2013, *Q*8 = −75.1182, *Q*9 = −72.4609, *Q*10 = −1709.8307, *Q*11 = 0.0108, *Q*12 = −0.2002, *Q*13 = 0.1463, and *Q*14 = −0.4453.

In both eqs 16 and 17 including the same descriptors, three points of specific charge density from the sigma profile (i.e., “*C*–0.018”, “*C*–0.011”, and “*C*0”) are the cationic descriptors, and nine points of specific charge density (i.e., “A–0.009”, “A0”, “A0.002”, “A0.003”, “A0.009”, “A0.010”, “A0.017′, “A0.020”, and “A0.029”) are the anionic descriptors. The values of probability at above points of specific charge density for each cation and anion are used for the prediction of γ_H_2___O_^∞^ in ILs. In these models, the effect of temperature on the γ_H_2___O_^∞^ in ILs has been computationally taken into account based on the van’t Hoff equation. Such considerations were lacking in the former studies (Gonfa et al.^1^ and Benimam et al.^63^). The values of the statistical parameters of each model are shown in Table 4.

**Table 4:** Values of Statistical Parameters of Selected QSPR Models for Both of Training and Validation Sets

eqs. no	sets	number of data points	*R*_2_	*R*_2_-Adj	*Q*^2^-LOO	*Q*^2^-LMO	F	S	RMSE	AARD %	AAD
(16)	training	297	0.99	0.99	0.99	0.98	1937	0.1411	0.1378	33	0.099
	validation	83	0.85						0.3344	30	0.283
(17)	training	285	0.98	0.98	0.98	0.98	1062	0.1935	0.1887	29	0.142
	validation	95	0.98						0.1796	29	0.139

As indicated in Table 4, the obtained values of *Q*^2^ (either LOO or LMO) for each developed model were extremely high which are confirming that each model has accurate capability for the prediction of γ_H_2___O_^∞^ in studied ILs in a wide range of temperatures (see Table 1). Also, the Y-scrambling technique has been carried out on the training set in QSARINS software for each selected QSAR/QSPR model, and results confirmed the validity of each model. As external validation, it is also shown that γ_H_2___O_^∞^ in ILs of validation set predicted with enough accuracy based on the obtained values of AAD and RMSE. In the LOIO–CV method (i.e., eq 16), the temperature dependency of many validation-set’s ILs has been predicted well at each triple-region. Such good consistency had not been observed in the literature.^1,63^

First, it should be mentioned that the obtained values of statistical parameters of this study were much better than those values which were reported for the test set by Gonfa et al.^1^ For example, the (*R*^2^ and AARD %) parameters of this study (i.e., eq 16) for the same test set [see Supporting Information Excel file (see Sheet S8)] which was previously^1^ specified, were obtained (0.97, 26). This enhancement can be attributed to two important points: (1) the selection of most important molecular descriptors and (2) diversity of cation and anion structures in the data set. It seemed that these two points were neglected by Gonfa et al.^1^ Another advantage of the proposed models in this study in comparison with Gonfa’s model is the wider AD. The more structural variations of cation and anion in the training set, the more AD. In other words, the developed QSAR/QSPR models in this study are more reliable than former models for the screening of ILs for systems containing H_2_O. Apart from the above advantages, the proposed QSAR/QSPR models in this study have completely solved the issue in the previous model (i.e., the positive coefficient of 1/*T*) in Gonfa’s study. As can be seen in eqs 16 and 17, the adjustable parameter of 1/*T* (in van’t Hoff equation) in each model varies with respect to the structure and nature of cations and anions and it is not a constant value. For this reason, the low region of γ_H_2___O_^∞^ was predicted excellent using eqs 16 and 17, unlike the Gonfa’s model. For instance, the values of γ_H_2___O_^∞^ in some ILs (i.e., [C4C1Im][TFA] and [C1C1Im][DMP]) in a wide range of temperatures have been increasingly predicted using eqs 16 and 17 with increasing temperature, while the proposed model by Gonfa et al.^1^ has decreasingly predicted them which were completely inverse with experimental results.

In comparison with Benimam et al.,^63^ it should be mentioned that they took advantage of some powerful nonlinear methods which are complicated for general use. In contrast, the developed MLR-QSPR models in our study are much easier. The model by Beniman et al.^63^ was examined only for imidazolium-based ILs, while our developed models were examined for many kinds of ILs. This point varies the extensive AD of our predictive models.

Therefore, the proposed QSAR/QSPR models (i.e., eqs 16 and 17) could take into account the effect of temperature on the γ_H_2___O_^∞^ in more than 95% of ILs (the included ILs in Table 1) well and satisfactorily. However, for some limited ILs like [C6C1Im][SCN], the capability of models (i.e., eqs 16 and 17) was not satisfactory. For example, the temperature dependence of γ_H_2___O_^∞^ in this IL was not correct. Such conflict was observed also by Thangarajoo et al.^3^ For example, the values of γ_MeOH_^∞^ in [HydEMIM][FAP] were experimentally increasing with increasing temperature, while the developed model using GCM by Thangarajoo et al.^3^ predicted the opposite.

**Figure 3:**
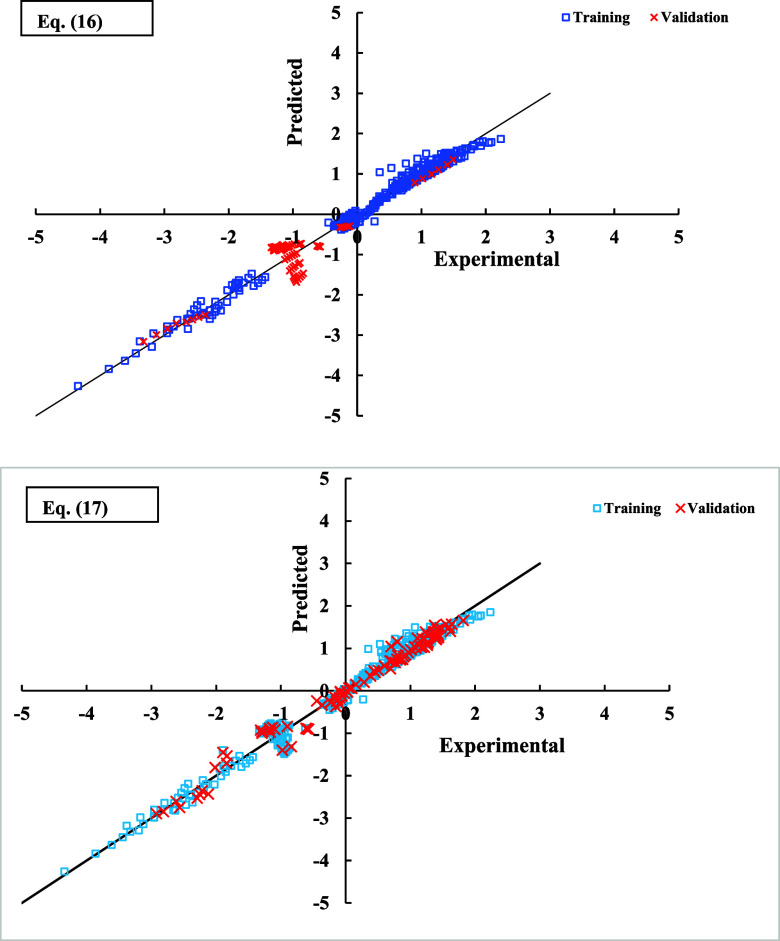
Predicted versus experimental values (ln-based) for both of training and validation sets using eqs 16 and 17.

The plots of predicted versus experimental values for both of training and validation sets, which were obtained using QSAR/QSPR models (i.e., eqs 16 and 17), are shown in Figure 3.

**Figure 4:**
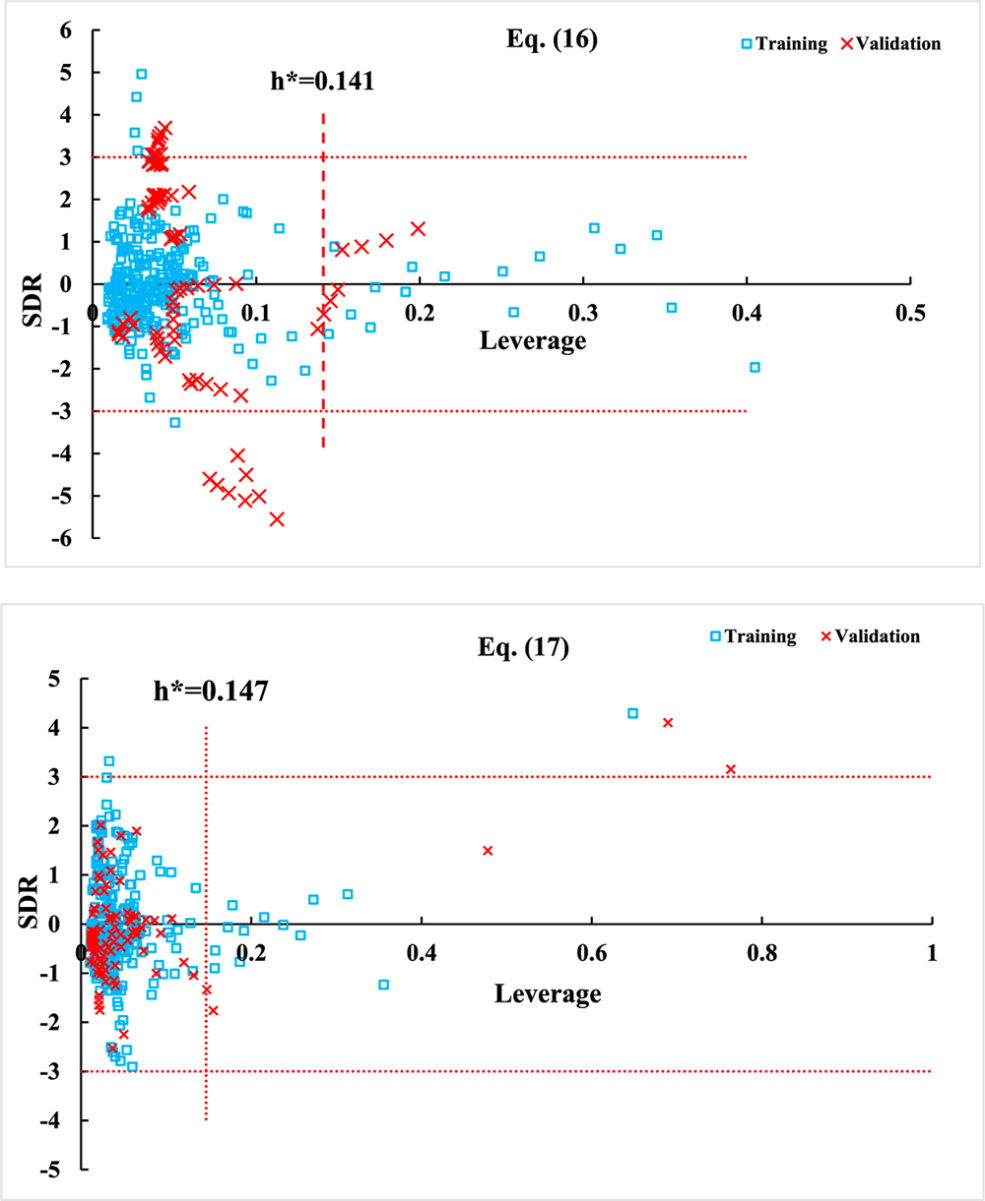
Williams plots for training and validation sets using eqs 16 and 17.

The Williams plots for the training and validation sets which were obtained using QSAR/QSPR models (i.e., eqs 16 and 17) are shown in Figure 4. According to these plots, except of three data points which seem to be outliers, the values of SRD for some data points are higher than ±3, but their leverage values are lower than h* which mean no outliers are in the data set.

As the temperature dependency of γ_H_2___O_^∞^ in ILs can be expressed by physicochemical properties (i.e., ΔH_IL_^E,∞^ and ΔS_IL_^E,∞^), it may be interesting to look at these predicted properties separately. Although these values are determined indirectly from the temperature dependency of γ_H_2___O_^∞^, they may reveal interesting further structure–property relationships. These two properties for each studied IL in Table 1 have been calculated using descriptors in “A” and “B” parameters and have been reported in Table 5. More details can be found in the Supporting Information Excel file (see Sheet S9).

**Table 5:** Calculated Partial Molar Excess Enthalpy (*i*.*e*., ΔH_IL_^E,∞^) and Entropy (*i*.*e*., ΔS_IL_^E,∞^) of Each IL at Infinite Dilution Using Molecular Descriptors in “*A*” and “*B*” Parameters of van’t Hoff Equation

mixture IL/H_2_O	temperature range (K)	range of ln (γ_H_2___O_^∞^_Exp_)	ΔH_IL_^E,∞^(J·(mol)^−1^)	ΔS_IL_^E,∞^(J·(K·mol)^−1^)
[C4C1Im][SCN]	328–368	(−1.298) – (−1.269)	–638.42	5.32
[C4–3-C1Py][CF3SO3]	318–358	(−0.116) – (−0.267)	667.88	4.57
[C4C1Pyrro][FAP]	318–368	(2.236) – (1.350)	8130.46	10.05
[C3C1Pip][NTF2]	308–358	(1.488) – (0.896)	10460.40	22.69
[HOC3Py][FAP]	308–358	(0.974) – (0.548)	6046.38	10.44
[C4C1Im][TFA]	333–393	(−1.851) – (−1.639)	6046.38	10.44
[C2C1Im][SCN]	328–368	(−1.298) – (−1.269)	–6402.96	–4.02
[C4C1Pip][SCN]	328–368	(−1.105) – (−1.064)	–642.39	5.56
[C4–4-C1Py][SCN]	328–368	(−1.187) – (−1.155)	–481.46	5.31
[C4C1Pyrro][SCN]	328–368	(−1.335) – (−1.269)	–743.70	5.18
[C6C1Im][SCN]	328–368	(−0.572) – (−0.623)	–475.03	5.42
[C6-iqui][SCN]	328–368	(−0.913) – (−0.872)	–627.75	4.81
[C4C1Mor][C(CN)3]	318–368	(0.029) – (−0.248)	–657.04	4.26
[C4C1Pyrro][C(CN)3]	318–368	(−0.027) – (−0.261)	3780.99	13.47
[C4C1Pyrro][B(CN)4]	318–368	(0.765) – (0.246)	4460.35	13.33
[C10C1Im][B(CN)4]	328–378	(0.751) – (0.300)	8595.49	21.32
[C2C1Im][B(CN)4]	298–358	(0.806) – (0.254)	8443.44	19.56
[C6C1Im][B(CN)4]	318–368	(0.717) – (0.285)	8428.13	21.46
[C4C1Pyrro][CF3SO3]	318–368	(−0.145) – (−0.339)	8442.78	20.71
[C4C1Im][CF3SO3]	328–368	(−0.212) – (−0.362)	966.03	4.78
[C2C1Im][TFA]	348–368	(−1.944) – (−1.838)	802.64	4.68
[C6OC1C1Im][NTF2]	298–358	(1.474) – (0.712)	–6406.92	–3.77
[(C6OCH2)2Im][NTF2]	298–368	(1.488) – (0.593)	10058.68	21.84
[C4–4-C1Py][NTF2]	298–368	(1.581) – (0.717)	9763.95	20.66
[N1112OH][NTF2]	318–368	(0.270) – (−0.069)	10210.83	22.40
[C8iquin][NTF2]	328–368	(1.398) – (0.838)	7813.72	23.27
[C4C1Pip][NTF2]	308–358	(1.566) – (0.879)	10473.06	22.52
[S222][NTF2]	308–368	(1.363) – (0.631)	10178.70	22.46
[C1C1Im][NTF2]	303–333	(1.050) – (0.698)	10265.26	23.10
[C2C1Im][NTF2]	293–323	(1.302) – (0.932)	10312.13	22.78
[C2Py][NTF2]	303–323	(1.022) – (0.693)	9957.94	22.84
[HOC3Py][NTF2]	318–378	(0.425) – (0.029)	8395.42	23.03
[COC2C1Pip][NTF2]	318–368	(1.208) – (0.625)	10367.70	22.74
[COC2C1Mor][NTF2]	318–368	(0.912) – (0.343)	9832.35	23.01
[COC2C1Pyrro][NTF2]	318–368	(1.147) – (0.615)	10349.27	22.86
[C5C1Pip][NTF2]	308–358	(1.619) – (0.892)	10470.42	22.32
[C6C1Pip][NTF2]	308–358	(1.669) – (0.896)	10484.03	22.06
[C4–4-C1Py][N(CN)2]	338–368	(−1.224) – (−1.158)	–2762.59	–1.23
[COC2C1Mor][FAP]	318–368	(1.547) – (1.101)	7483.32	10.43
[COC2C1Pip][FAP]	318–368	(1.954) – (1.308)	8018.66	10.15
[COC2C1Pyrro][FAP]	318–368	(2.086) – (1.371)	8000.23	10.28
[HOC2C1Im][FAP]	318–368	(0.797) – (0.570)	5809.05	10.37
[C1C1Im][DMP]	363–383	(−2.960) – (−2.856)	–9679.53	–2.11
[C4C1Im][Br]	333–393	(−2.631) – (−2.017)	–17394.13	–28.59
[C2C1Im][MeSO3]	318–358	(−2.645) – (−2.430)	–10297.05	–10.80
[C2C1Im][C(CN)3]	318–368	(−0.080) – (−0.153)	4292.99	13.47
[C2C1Im][FAP]	318–368	(1.906) – (1.425)	7963.10	10.19
[HOC2C1Im][N(CN)2]	328–358	(−0.946) – (−0.991)	–4815.33	–0.67
[C4C1Im][Ac]	298–393	(−4.342) – (−3.194)	–10035.54	1.80
[C4C1Im][C(CN)3]	318–368	(0.019) – (−0.164)	4296.96	13.22
[C4C1Im][Cl]	333–428	(−3.324) – (−2.364)	–8171.56	1.73
[C4C1Im][N(CN)2]	318–368	(−1.171) – (−0.978)	–2657.32	–1.10
[C4C1Im][NTF2]	293–363	(1.373) – (0.688)	10316.10	22.53
[C4C1Im][DMP]	333–428	(−3.381) – (−2.292)	–9628.69	–2.68
[C4C1Im][MeSO3]	333–428	(−2.120) – (−1.431)	–10293.08	–11.05
[C6C1Im][CF3SO3]	303–333	(0.270) – (−0.446)	813.32	4.17
[B Cya Py][NTF2]	308–368	(1.196) – (0.693)	10172.84	22.71
[C12C1Im][NTF2]	318–368	(1.501) – (1.022)	10334.24	20.30
[C4C1Py][C(CN)3]	318–368	(−0.029) – (−0.228)	4191.69	13.09
[4OHC3 4-C1Mor][NTF2]	318–368	(0.357) – (0.095)	8397.84	23.15
[C8Quin][NTF2]	313–353	(1.071) – (0.350)	10634.38	21.47
[C6Quin][NTF2]	313–353	(1.446) – (1.098)	10627.95	22.04
[P4442][DEP]	328–368	(−2.292) – (−2.154)	–8271.31	–3.62
[C8C1Im][NTF2]	303–333	(1.358) – (0.947)	10302.30	21.46
[C2C1Im][C8SO4]	333–358	(−1.832) – (−1.897)	–6116.82	–2.60
[C2C1Mor][N(CN)2]	318–368	(−1.127) – (−0.941)	–3166.68	–0.59
[HOC3Py][N(CN)2]	328–358	(−0.918) – (−0.839)	–4578.00	–0.60
[OHC3–C1Im][N(CN)2]	318–368	(−1.049) – (−0.891)	–3928.65	–0.68

**Figure 5:**
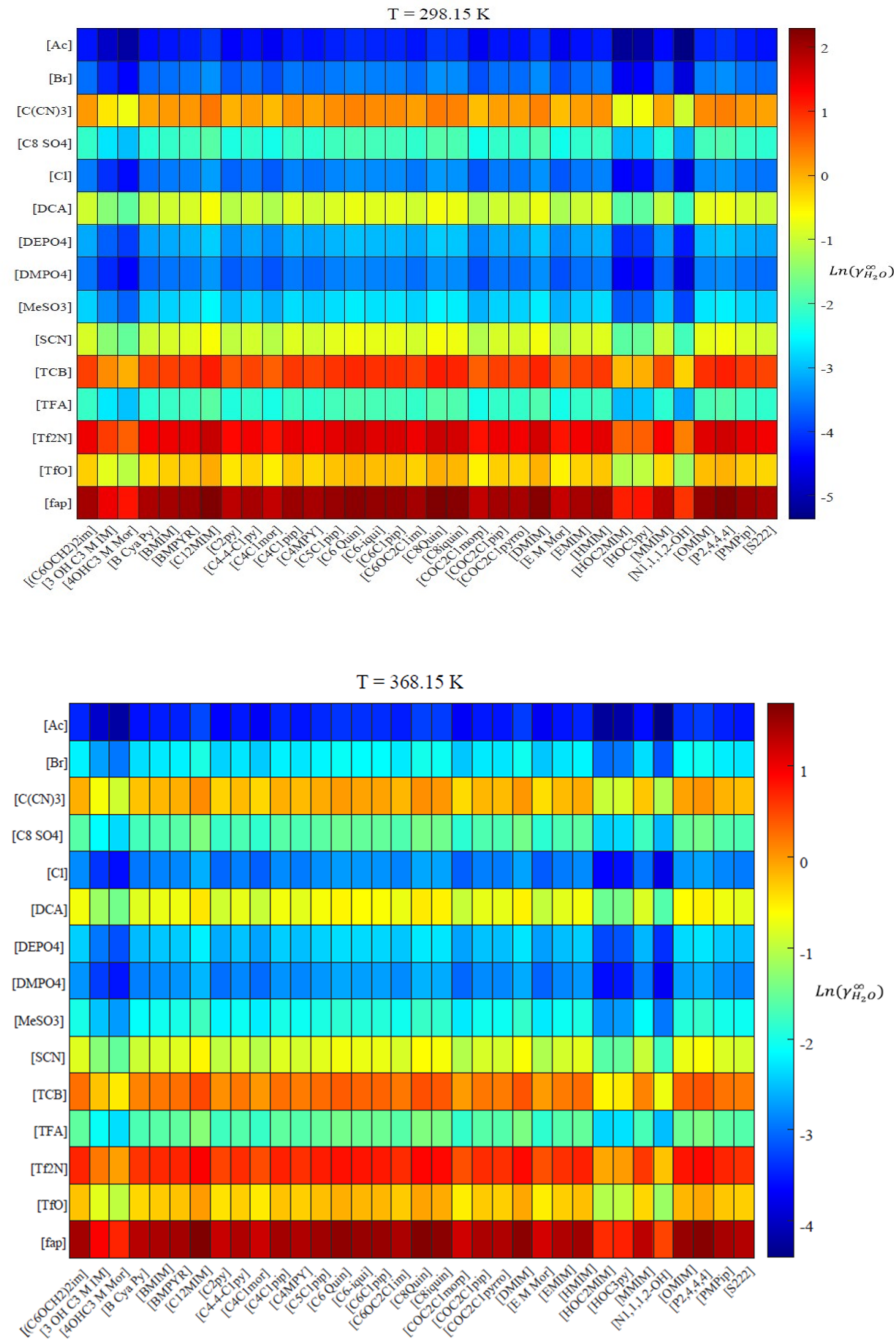
Predicted ln γ_H_2___O_^∞^ values by the QSPR-model eq 16 at (a) 298 K and (b) 368 K.

Based on the proposed QSAR/QSPR models, a large list including values of ln γ_H_2___O_^∞^-Pseudo-Exp in large numbers of none-studied ILs at different temperatures has been provided in the Supporting Information Excel file (see Sheet S10). Also, to ease and facilitate the IL-screening for those systems including H_2_O, separate heatmaps have been provided for two different temperatures, as shown in Figure 5. According to these heatmaps, the ILs with highest and lowest affinities to H_2_O can be found. As can be seen in Figure 5, anions have a much larger effect than the cations. For example, when anion is constant, the color bands are very similar independent of the cation.

## 4 Conclusions

The developed QSAR/QSPR models could successfully predict γ_H_2___O_^∞^ in ILs as a function of temperature. The versatility of the models is displayed by the ability to predict the effect of temperature both the increase and decrease of the γ_H_2___O_^∞^ which is determined by the structure of the IL. The present models succeed to predict the temperature dependency of γ_H_2___O_^∞^ for new ILs.

In this study, the γ_H_2___O_^∞^ in large data set of ILs has been predicted with a very good accuracy using QSPR-based COSMO-RS descriptors. The obtained values of statistical parameters (RMSE, AAD, *R*^2^, and *Q*^2^_LOO–CV_) of the developed QSPR model were acceptable for either training or validation set. The obtained values of the RMSE parameter were 0.1378 and 0.3344 for training and validation sets, respectively, expressing the high prediction capability of the QSPR model for the studied data set. The internal and LOIO–CV-external validations verified that the prediction of γ_H_2___O_^∞^ in a huge number of ILs which had not been experimentally studied can be practical with respect to the leverage value of ILs and AD of the QSPR model.

## Data Availability

The used experimental data for the training of the QSPR models can be found in the Supporting Information Excel file. In this study, the sigma-profile descriptors were taken from COSMO-RS software and model development has been performed in QSARINS free-software.

## Supporting Information Available

Three regions, cation and anion matrix, data points of data set, results, training and validation sets, Gonfa’s test set, thermodynamic properties, pseudo-experimental data, and nomenclature of cations and anions and smile files (XLSX)

## References

[ref1] Gonfa G.; Bustam M. A.; Shariff A. M.; Muhammad N.; Ullah S. Quantitative structure–activity relationships (QSARs) for estimation of activity coefficient at infinite dilution of water in ionic liquids for natural gas dehydration. J. Taiwan Inst. Chem. Eng. 2016, 66, 222–229. 10.1016/j.jtice.2016.06.027.

[ref2] Gonfa G.; Bustam M. A.; Sharif A. M.; Mohamad N.; Ullah S. Tuning ionic liquids for natural gas dehydration using COSMO-RS methodology. J. Nat. Gas Sci. Eng. 2015, 27, 1141–1148. 10.1016/j.jngse.2015.09.062.

[ref3] Thangarajoo N.; Matheswaran P.; Johari K.; Kurnia K. A. Overview of Activity Coefficient of Methanol at Infinite Dilution in Ionic Liquids and their Modeling using Group Contribution Model. J. Chem. Eng. Data 2019, 64 (4), 1760–1769. 10.1021/acs.jced.8b01246.

[ref4] Domańska U.; Marciniak A. Activity coefficients at infinite dilution measurements for organic solutes and water in the ionic liquid 4-methyl-N-butyl-pyridinium bis (trifluoromethylsulfonyl)-imide. J. Chem. Thermodyn. 2009, 41 (12), 1350–1355. 10.1016/j.jct.2009.06.011.

[ref5] Li Y.; Wang L. S.; Li M. Y.; Tian N. N. Activity Coefficients at Infinite Dilution of Organic Solutes in 1-Decyl-3-methylimidazolium Tetrafluoroborate Using Gas– Liquid Chromatography. J. Chem. Eng. Data 2011, 56 (4), 1704–1708. 10.1021/je100952p.

[ref6] Domańska U.; Laskowska M. Measurements of activity coefficients at infinite dilution of aliphatic and aromatic hydrocarbons, alcohols, thiophene, tetrahydrofuran, MTBE, and water in ionic liquid [BMIM][SCN] using GLC. J. Chem. Thermodyn. 2009, 41 (5), 645–650. 10.1016/j.jct.2008.12.018.

[ref7] Marciniak A.; Wlazlo M. Activity coefficients at infinite dilution measurements for organic solutes and water in the ionic liquid 1-butyl-3-methyl-pyridinium trifluoromethanesulfonate. J. Chem. Eng. Data 2010, 55 (9), 3208–3211. 10.1021/je1000582.

[ref8] Domańska U.; Lukoshko E. V.; Królikowski M. Measurements of activity coefficients at infinite dilution for organic solutes and water in the ionic liquid 1-butyl-1-methylpyrrolidinium tris (pentafluoroethyl) trifluorophosphate ([BMPYR][FAP]). Chem. Eng. J. 2012, 183, 261–270. 10.1016/j.cej.2011.12.072.

[ref9] Domańska U.; Paduszyński K. Measurements of activity coefficients at infinite dilution of organic solutes and water in 1-propyl-1-methylpiperidinium bis {(trifluoromethyl) sulfonyl} imide ionic liquid using glc. J. Chem. Thermodyn. 2010, 42 (11), 1361–1366. 10.1016/j.jct.2010.05.017.

[ref10] Marciniak A.; Wlazło M. Activity coefficients at infinite dilution measurements for organic solutes and water in the ionic liquid 1-(3-hydroxypropyl) pyridinium trifluorotris (perfluoroethyl) phosphate. J. Phys. Chem. B 2010, 114 (20), 6990–6994. 10.1021/jp101573f. 20429540

[ref11] Khan I.; Kurnia K. A.; Mutelet F.; Pinho S. P.; Coutinho J. A. Probing the interactions between ionic liquids and water: experimental and quantum chemical approach. J. Phys. Chem. B 2014, 118 (7), 1848–1860. 10.1021/jp4113552. 24467614

[ref12] Domańska U.; Marciniak A. Measurements of activity coefficients at infinite dilution of aromatic and aliphatic hydrocarbons, alcohols, and water in the new ionic liquid [EMIM][SCN] using GLC. J. Chem. Thermodyn. 2008, 40 (5), 860–866. 10.1016/j.jct.2008.01.004.

[ref13] Domanska U.; Królikowska M. Measurements of activity coefficients at infinite dilution for organic solutes and water in the ionic liquid 1-butyl-1-methylpiperidinium thiocyanate. J. Chem. Eng. Data 2011, 56 (1), 124–129. 10.1021/je101008y.

[ref14] Domanska U.; Królikowska M. Measurements of activity coefficients at infinite dilution in solvent mixtures with thiocyanate-based ionic liquids using GLC technique. J. Phys. Chem. B 2010, 114 (25), 8460–8466. 10.1021/jp103496d. 20527942

[ref15] Domańska U.; Marciniak A.; Królikowska M.; Arasimowicz M. Activity coefficients at infinite dilution measurements for organic solutes and water in the ionic liquid 1-hexyl-3-methylimidazolium thiocyanate. J. Chem. Eng. Data 2010, 55 (7), 2532–2536. 10.1021/je900890u.

[ref16] Królikowska M.; Karpińska M.; Królikowski M. Measurements of activity coefficients at infinite dilution for organic solutes and water in N-hexylisoquinolinium thiocyanate,[HiQuin][SCN] using GLC. J. Chem. Thermodyn. 2013, 62, 1–7. 10.1016/j.jct.2013.02.004.

[ref17] Domańska U.; Lukoshko E. V. Thermodynamics and activity coefficients at infinite dilution for organic solutes and water in the ionic liquid 1-butyl-1-methylmorpholinium tricyanomethanide. J. Chem. Thermodyn. 2014, 68, 53–59. 10.1016/j.jct.2013.08.030.

[ref18] Domańska U.; Lukoshko E. V. Measurements of activity coefficients at infinite dilution for organic solutes and water in the ionic liquid 1-butyl-1-methylpyrrolidinium tricyanomethanide. J. Chem. Thermodyn. 2013, 66, 144–150. 10.1016/j.jct.2013.07.004.

[ref19] Domańska U.; Królikowski M.; Acree Jr W. E. Thermodynamics and activity coefficients at infinite dilution measurements for organic solutes and water in the ionic liquid 1-butyl-1-methylpyrrolidinium tetracyanoborate. J. Chem. Thermodyn. 2011, 43 (12), 1810–1817. 10.1016/j.jct.2011.06.007.

[ref20] Domańska U.; Marciniak A. Physicochemical properties and activity coefficients at infinite dilution for organic solutes and water in the ionic liquid 1-decyl-3-methylimidazolium tetracyanoborate. J. Phys. Chem. B 2010, 114 (49), 16542–16547. 10.1021/jp109469s. 21090701

[ref21] Domańska U.; Królikowska M.; Acree Jr W. E.; Baker G. A. Activity coefficients at infinite dilution measurements for organic solutes and water in the ionic liquid 1-ethyl-3-methylimidazolium tetracyanoborate. J. Chem. Thermodyn. 2011, 43 (7), 1050–1057. 10.1016/j.jct.2011.02.012.

[ref22] Domańska U.; Lukoshko E. V.; Wlazło M. Measurements of activity coefficients at infinite dilution for organic solutes and water in the ionic liquid 1-hexyl-3-methylimidazolium tetracyanoborate. J. Chem. Thermodyn. 2012, 47, 389–396. 10.1016/j.jct.2011.11.025.

[ref23] Domańska U.; Redhi G. G.; Marciniak A. Activity coefficients at infinite dilution measurements for organic solutes and water in the ionic liquid 1-butyl-1-methylpyrrolidinium trifluoromethanesulfonate using GLC. Fluid Phase Equilib. 2009, 278 (1–2), 97–102. 10.1016/j.fluid.2009.01.011.

[ref24] Domanska U.; Marciniak A. Activity coefficients at infinite dilution measurements for organic solutes and water in the ionic liquid 1-butyl-3-methylimidazolium trifluoromethanesulfonate. J. Phys. Chem. B 2008, 112 (35), 11100–11105. 10.1021/jp804107y. 18693694

[ref25] Domańska U.; Marciniak A. Activity coefficients at infinite dilution measurements for organic solutes and water in the ionic liquid 1-ethyl-3-methylimidazolium trifluoroacetate. J. Phys. Chem. B 2007, 111 (41), 11984–11988. 10.1021/jp075079+. 17887791

[ref26] Domańska U.; Marciniak A. Activity coefficients at infinite dilution measurements for organic solutes and water in the 1-hexyloxymethyl-3-methyl-imidazolium and 1, 3-dihexyloxymethyl-imidazolium bis (trifluoromethylsulfonyl)-imide ionic liquids—The cation influence. Fluid Phase Equilib. 2009, 286 (2), 154–161. 10.1016/j.fluid.2009.08.017.

[ref27] Domańska U.; Papis P.; Szydłowski J. Thermodynamics and activity coefficients at infinite dilution for organic solutes, water and diols in the ionic liquid choline bis (trifluoromethylsulfonyl) imide. J. Chem. Thermodyn. 2014, 77, 63–70. 10.1016/j.jct.2014.04.024.

[ref28] Domańska U.; Zawadzki M.; Królikowska M.; Marc Tshibangu M.; Ramjugernath D.; Letcher T. M. Measurements of activity coefficients at infinite dilution of organic compounds and water in isoquinolinium-based ionic liquid [C_8_iQuin] [NTf_2_] using GLC. J. Chem. Thermodyn. 2011, 43 (3), 499–504. 10.1016/j.jct.2010.10.026.

[ref29] Paduszynski K.; Domanska U. Limiting activity coefficients and gas–liquid partition coefficients of various solutes in piperidinium ionic liquids: measurements and LSER calculations. J. Phys. Chem. B 2011, 115 (25), 8207–8215. 10.1021/jp202010w. 21634373

[ref30] Domańska U.; Marciniak A. Activity coefficients at infinite dilution measurements for organic solutes and water in the ionic liquid triethylsulphonium bis (trifluoromethylsulfonyl) imide. J. Chem. Thermodyn. 2009, 41 (6), 754–758. 10.1016/j.jct.2008.12.005.

[ref31] Krummen M.; Wasserscheid P.; Gmehling J. Measurement of activity coefficients at infinite dilution in ionic liquids using the dilutor technique. J. Chem. Eng. Data 2002, 47 (6), 1411–1417. 10.1021/je0200517.

[ref32] Kato R.; Gmehling J. Activity coefficients at infinite dilution of various solutes in the ionic liquids [MMIM]+[CH3SO4]–,[MMIM]+[CH3OC2H4SO4]–,[MMIM]+[(CH3) 2PO4]–,[C5H5NC2H5]+[(CF3SO2) 2N]– and [C5H5NH]+[C2H5OC2H4OSO3]. Fluid Phase Equilib. 2004, 226, 37–44. 10.1016/j.fluid.2004.08.039.

[ref33] Marciniak A. Activity coefficients at infinite dilution and physicochemical properties for organic solutes and water in the ionic liquid 1-(3-hydroxypropyl) pyridinium bis (trifluoromethylsulfonyl)-amide. J. Chem. Thermodyn. 2011, 43 (10), 1446–1452. 10.1016/j.jct.2011.04.018.

[ref34] Marciniak A.; Wlazło M. Activity coefficients at infinite dilution and physicochemical properties for organic solutes and water in the ionic liquid 1-(2-methoxyethyl)-1-methylpiperidinium bis (trifluoromethylsulfonyl)-amide. J. Chem. Thermodyn. 2012, 49, 137–145. 10.1016/j.jct.2012.01.019.

[ref35] Marciniak A.; Wlazło M. Activity coefficients at infinite dilution and physicochemical properties for organic solutes and water in the ionic liquid 4-(2-methoxyethyl)-4-methylmorpholinium bis (trifluoromethylsulfonyl)-amide. J. Chem. Thermodyn. 2012, 47, 382–388. 10.1016/j.jct.2011.11.021.

[ref36] Marciniak A.; Wlazło M. Activity coefficients at infinite dilution and physicochemical properties for organic solutes and water in the ionic liquid 1-(2-methoxyethyl)-1-methylpyrrolidinium bis(trifluoromethylsulfonyl)-amide. J. Chem. Thermodyn. 2012, 54, 90–96. 10.1016/j.jct.2012.03.015.

[ref37] Paduszyński K.; Domańska U. Experimental and theoretical study on infinite dilution activity coefficients of various solutes in piperidinium ionic liquids. J. Chem. Thermodyn. 2013, 60, 169–178. 10.1016/j.jct.2013.01.005.

[ref38] Królikowski M.; Królikowska M. The study of activity coefficients at infinite dilution for organic solutes and water in 1-butyl-4-methylpyridinium dicyanamide,[B4MPy][DCA] using GLC. J. Chem. Thermodyn. 2014, 68, 138–144. 10.1016/j.jct.2013.09.007.

[ref39] Wlazło M.; Marciniak A. Activity coefficients at infinite dilution and physicochemical properties for organic solutes and water in the ionic liquid 4-(2-methoxyethyl)-4-methylmorpholinium trifluorotris (perfluoroethyl) phosphate. J. Chem. Thermodyn. 2012, 54, 366–372. 10.1016/j.jct.2012.05.017.

[ref40] Marciniak A.; Wlazło M. Activity coefficients at infinite dilution and physicochemical properties for organic solutes and water in the ionic liquid 1-(2-methoxyethyl)-1-methylpiperidinium trifluorotris(perfluoroethyl)phosphate. J. Chem. Thermodyn. 2013, 57, 197–202. 10.1016/j.jct.2012.08.016.

[ref41] Marciniak A.; Wlazło M. Activity coefficients at infinite dilution and physicochemical properties for organic solutes and water in the ionic liquid 1-(2-methoxyethyl)-1-methylpyrrolidinium trifluorotris (perfluoroethyl) phosphate. J. Chem. Thermodyn. 2013, 60, 57–62. 10.1016/j.jct.2013.01.007.

[ref42] Marciniak A.; Wlazło M. Activity coefficients at infinite dilution and physicochemical properties for organic solutes and water in the ionic liquid 1-(2-hydroxyethyl)-3-methylimidazolium trifluorotris (perfluoroethyl) phosphate. J. Chem. Thermodyn. 2013, 64, 114–119. 10.1016/j.jct.2013.05.008. PMC4412692 25960582

[ref43] Domańska U.; Królikowski M. Measurements of activity coefficients at infinite dilution for organic solutes and water in the ionic liquid 1-ethyl-3-methylimidazolium methanesulfonate. J. Chem. Thermodyn. 2012, 54, 20–27. 10.1016/j.jct.2012.03.005.

[ref44] Karpińska M.; Wlazło M.; Domańska U. Separation of binary mixtures based on gamma infinity data using [EMIM][TCM] ionic liquid and modelling of thermodynamic functions. J. Mol. Liq. 2017, 225, 382–390. 10.1016/j.molliq.2016.11.081.

[ref45] Wlazło M.; Marciniak A.; Letcher T. M. Activity coefficients at infinite dilution and physicochemical properties for organic solutes and water in the ionic liquid 1-ethyl-3-methylimidazolium trifluorotris (perfluoroethyl) phosphate. J. Solution Chem. 2015, 44, 413–430. 10.1007/s10953-014-0274-0. 25960582 PMC4412692

[ref46] Paduszyński K.; Królikowska M. Effect of side chain functional group on interactions in ionic liquid systems: insights from infinite dilution thermodynamic data. J. Phys. Chem. B 2017, 121 (43), 10133–10145. 10.1021/acs.jpcb.7b08797. 28976771

[ref47] Lukoshko E.; Mutelet F.; Domanska U. Experimental and theoretically study of interaction between organic compounds and tricyanomethanide based ionic liquids. J. Chem. Thermodyn. 2015, 85, 49–56. 10.1016/j.jct.2014.12.027.

[ref48] Martins M. A.; Coutinho J. A.; Pinho S. P.; Domańska U. Measurements of activity coefficients at infinite dilution of organic solutes and water on polar imidazolium-based ionic liquids. J. Chem. Thermodyn. 2015, 91, 194–203. 10.1016/j.jct.2015.07.042.

[ref49] Domańska U.; Wlazło M.; Karpińska M. Activity coefficients at infinite dilution of organic solvents and water in 1-butyl-3-methylimidazolium dicyanamide. A literature review of hexane/hex-1-ene separation. Fluid Phase Equilib. 2016, 417, 50–61. 10.1016/j.fluid.2016.02.004.

[ref50] Singh S.; Bahadur I.; Naidoo P.; Redhi G.; Ramjugernath D. Application of 1-butyl-3-methylimidazolium bis (trifluoromethylsulfonyl) imide ionic liquid for the different types of separations problem: Activity coefficients at infinite dilution measurements using gas-liquid chromatography technique. J. Mol. Liq. 2016, 220, 33–40. 10.1016/j.molliq.2016.04.059.

[ref51] Liebert V.; Nebig S.; Gmehling J. Experimental and predicted phase equilibria and excess properties for systems with ionic liquids. Fluid Phase Equilib. 2008, 268 (1–2), 14–20. 10.1016/j.fluid.2008.03.011.

[ref52] Wlazło M.; Karpińska M.; Domańska U. A 1-alkylcyanopyridinium-based ionic liquid in the separation processes. J. Chem. Thermodyn. 2016, 97, 253–260. 10.1016/j.jct.2016.01.017.

[ref53] Domańska U.; Wlazło M. Thermodynamics and limiting activity coefficients measurements for organic solutes and water in the ionic liquid 1-dodecyl-3-methylimidzolium bis (trifluoromethylsulfonyl) imide. J. Chem. Thermodyn. 2016, 103, 76–85. 10.1016/j.jct.2016.08.008.

[ref54] Wlazło M.; Marciniak A.; Zawadzki M.; Dudkiewicz B. Activity coefficients at infinite dilution and physicochemical properties for organic solutes and water in the ionic liquid 4-(3-hydroxypropyl)-4-methylmorpholinium bis (trifluoromethylsulfonyl)-amide. J. Chem. Thermodyn. 2015, 86, 154–161. 10.1016/j.jct.2015.02.024.

[ref55] Ayad A.; Mutelet F.; Negadi A.; Acree W. E. Jr; Jiang B.; Lu A.; Wagle D. V.; Baker G. A. Activity coefficients at infinite dilution for organic solutes dissolved in two 1-alkylquinuclidinium bis (trifluoromethylsulfonyl) imides bearing alkyl side chains of six and eight carbons. J. Mol. Liq. 2016, 215, 176–184. 10.1016/j.molliq.2015.12.029.

[ref56] Królikowska M.; Orawiec M. Activity Coefficients at Infinite Dilution of Organic Solutes and Water in Tributylethylphosphonium Diethylphosphate Using Gas–Liquid Chromatography: Thermodynamic Properties of Mixtures Containing Ionic Liquids. J. Chem. Eng. Data 2016, 61 (5), 1793–1802. 10.1021/acs.jced.5b00980.

[ref57] Kato R.; Gmehling J. Systems with ionic liquids: Measurement of VLE and γ∞ data and prediction of their thermodynamic behavior using original UNIFAC, mod. UNIFAC (Do) and COSMO-RS (Ol). J. Chem. Thermodyn. 2005, 37 (6), 603–619. 10.1016/j.jct.2005.04.010.

[ref58] Bahadur I.; Naidoo M.; Naidoo P.; Ramdath S.; Ramjugernath D.; Ebenso E. E. Screening of environmental friendly ionic liquid as a solvent for the different types of separations problem: Insight from activity coefficients at infinite dilution measurement using (gas+ liquid) chromatography technique. J. Chem. Thermodyn. 2016, 92, 35–42. 10.1016/j.jct.2015.08.017.

[ref59] Domańska U.; Karpińska M.; Wlazło M. Thermodynamic study of molecular interaction-selectivity in separation processes based on limiting activity coefficients. J. Chem. Thermodyn. 2018, 121, 112–120. 10.1016/j.jct.2018.02.014.

[ref60] Domańska U.; Wlazło M.; Karpińska M.; Zawadzki M. Separation of binary mixtures hexane/hex-1-ene, cyclohexane/cyclohexene and ethylbenzene/styrene based on limiting activity coefficients. J. Chem. Thermodyn. 2017, 110, 227–236. 10.1016/j.jct.2017.03.004.

[ref61] Karpińska M.; Wlazło M.; Zawadzki M.; Domańska U. Separation of binary mixtures hexane/hex-1-ene, cyclohexane/cyclohexene and ethylbenzene/styrene based on gamma infinity data measurements. J. Chem. Thermodyn. 2018, 118, 244–254. 10.1016/j.jct.2017.11.017.

[ref62] Revelli A. L.; Mutelet F.; Jaubert J. N.; Garcia-Martinez M.; Sprunger L. M.; Acree Jr W. E.; Baker G. A. Study of ether-, alcohol-, or cyano-functionalized ionic liquids using inverse gas chromatography. J. Chem. Eng. Data 2010, 55 (7), 2434–2443. 10.1021/je900838a.

[ref63] Benimam H.; Moussa C. S.; Hentabli M.; Hanini S.; Laidi M. Dragonfly-support vector machine for regression modeling of the activity coefficient at infinite dilution of solutes in imidazolium ionic liquids using σ-profile descriptors. J. Chem. Eng. Data 2020, 65 (6), 3161–3172. 10.1021/acs.jced.0c00168.

[ref64] Gorji A. E.; Sobati M. A.; Alopaeus V.; Uusi-Kyyny P. Toward solvent screening in the extractive desulfurization using ionic liquids: QSPR modeling and experimental validations. Fuel 2021, 302, 121159 10.1016/j.fuel.2021.121159.

[ref65] Gorji A. E.; Sobati M. A. Toward molecular modeling of thiophene distribution between the ionic liquid and hydrocarbon phases: Effect of hydrocarbon structure. J. Mol. Liq. 2019, 287, 110976 10.1016/j.molliq.2019.110976.

[ref66] Gorji A. E.; Sobati M. A. How anion structures can affect the thiophene distribution between imidazolium-based ionic liquid and hydrocarbon phases? A theoretical QSPR study. Energy Fuels 2019, 33 (9), 8576–8587. 10.1021/acs.energyfuels.9b02416.

[ref67] Gorji A. E.; Sobati M. A. Effect of the cation structure on the thiophene distribution between the ionic liquid with NTf2 anion and the hydrocarbon rich phases: A QSPR study. J. Mol. Liq. 2020, 313, 113551 10.1016/j.molliq.2020.113551.

[ref68] Gramatica P. Principles of QSAR modeling: comments and suggestions from personal experience. Int. J. Quant. Struct.-Prop. Relat. 2020, 5 (3), 61–97. 10.4018/IJQSPR.20200701.oa1.

[ref69] Gramatica P.; Sangion A. A historical excursus on the statistical validation parameters for QSAR models: a clarification concerning metrics and terminology. J. Chem. Inf. Model. 2016, 56 (6), 1127–1131. 10.1021/acs.jcim.6b00088. 27218604

[ref70] Gramatica P.; Chirico N.; Papa E.; Cassani S.; Kovarich S. QSARINS: A new software for the development, analysis, and validation of QSAR MLR models. J. Comput. Chem. 2013, 34, 2121–2132. 10.1002/jcc.23361.

[ref71] Diedenhofen M.; Eckert F.; Klamt A. Prediction of infinite dilution activity coefficients of organic compounds in ionic liquids using COSMO-RS. J. Chem. Eng. Data 2003, 48 (3), 475–479. 10.1021/je025626e.

[ref72] Klamt A. Conductor-like screening model for real solvents: a new approach to the quantitative calculation of solvation phenomena. J. Phys. Chem. 1995, 99 (7), 2224–2235. 10.1021/j100007a062.

[ref73] Klamt A.; Jonas V.; Bürger T.; Lohrenz J. C. Refinement and parametrization of COSMO-RS. J. Phys. Chem. A 1998, 102 (26), 5074–5085. 10.1021/jp980017s.

[ref74] Eckert F.; Klamt A. Fast solvent screening via quantum chemistry: COSMO-RS approach. AIChE J. 2002, 48 (2), 369–385. 10.1002/aic.690480220.

[ref75] BIOVIA COSMOtherm, Release; Dassault Systèmes, 2023 http://www.3ds.com.

[ref76] Modarresi H.; Modarress H.; Dearden J. C. QSPR model of Henry’s law constant for a diverse set of organic chemicals based on genetic algorithm-radial basis function network approach. Chemosphere 2007, 66 (11), 2067–2076. 10.1016/j.chemosphere.2006.09.049. 17113627

[ref77] Shahlaei M. Descriptor selection methods in quantitative structure–activity relationship studies: a review study. Chem. Rev. 2013, 113 (10), 8093–8103. 10.1021/cr3004339. 23822589

[ref78] Khooshechin S.; Dashtbozorgi Z.; Golmohammadi H.; Acree Jr W. E. QSPR prediction of gas-to-ionic liquid partition coefficient of organic solutes dissolved in 1-(2-hydroxyethyl)-1-methylimidazolium tris (pentafluoroethyl) trifluorophosphate using the replacement method and support vector regression. J. Mol. Liq. 2014, 196, 43–51. 10.1016/j.molliq.2014.03.012.

[ref79] Holland J. H. Adaption in Natural and Artificial Systems; The University of MichiganPress: Ann Arbor MI, 1975.

[ref80] Haupt R. L.; Haupt S. E. Practical Genetic Algorithms, 2nd ed.; John Wiley & Sons, Inc.: Hoboken, NJ, 2004.

[ref81] Lemaoui T.; Darwish A. S.; Hammoudi N. E. H.; Abu Hatab F.; Attoui A.; Alnashef I. M.; Benguerba Y. Prediction of electrical conductivity of deep eutectic solvents using COSMO-RS sigma profiles as molecular descriptors: a quantitative structure–property relationship study. Ind. Eng. Chem. Res. 2020, 59 (29), 13343–13354. 10.1021/acs.iecr.0c02542.

[ref82] Ojha P. K.; Roy K. Comparative QSARs for antimalarial endochins: importance of descriptor-thinning and noise reduction prior to feature selection. Chemom. Intell. Lab. Syst. 2011, 109 (2), 146–161. 10.1016/j.chemolab.2011.08.007.

[ref83] He W.; Yan F.; Jia Q.; Xia S.; Wang Q. Description of the thermal conductivity λ (T, P) of ionic liquids using the structure–property relationship method. J. Chem. Eng. Data 2017, 62 (8), 2466–2472. 10.1021/acs.jced.7b00422.

[ref84] Amini Z.; Fatemi M. H.; Gharaghani S. Hybrid docking-QSAR studies of DPP-IV inhibition activities of a series of aminomethyl-piperidones. Comput. Biol. Chem. 2016, 64, 335–345. 10.1016/j.compbiolchem.2016.08.003. 27570070

[ref85] Liu X.; Yu M.; Jia Q.; Yan F.; Zhou Y. N.; Wang Q. Leave-one-ion-out cross-validation for assisting in developing robust QSPR models of ionic liquids. J. Mol. Liq. 2023, 388, 122711 10.1016/j.molliq.2023.122711.

